# COVID-19 Vaccine Impacts in Saudi Arabia: A Cross-Sectional Study

**DOI:** 10.7759/cureus.40460

**Published:** 2023-06-15

**Authors:** Walaa Mohammedsaeed, Hanan Alrashidi, Sultan M Alsharif, Omaymah Aljardi, Asmaa Al-Sehli

**Affiliations:** 1 Medical Laboratory Technology Department, Faculty of Applied Medical Science, Taibah University, Madinah, SAU; 2 Biology Department, Faculty of Science, Taibah University, Madinah, SAU; 3 Family Medicine Department, Prince Mohammed Bin Abdulaziz Hospital, Madinah, SAU

**Keywords:** adverse effects, long-term, short-term, post-vaccination, covid-19

## Abstract

Background and purpose

Vaccinations provide disease protection through a variety of mechanisms; however, vaccines can occasionally cause adverse effects. Therefore, the objective of this investigation was to assess the short- and long-term adverse effects of COVID-19 vaccinations in Saudi Arabia.

Methods

In Saudi Arabia, between October 2021 and October 2022, a cross-sectional study was conducted. A validated Arabic and English dual-language online questionnaire were utilized to collect data.

Results

This investigation included 492 participants who were all immunized against the COVID-19 virus. There were 152 (30.9%) male participants and 340 (69.1%) female participants, with a mean age of 35±10.7 years. About 72.2% of study participants received three doses of vaccine, with 75.6% receiving the Pfizer-BioNTech vaccine, 22.2% receiving the Oxford-AstraZeneca vaccine, and 2.2% receiving the Moderna vaccine. Fever was observed in 66.3% (326/492) of the participants following vaccination, headache in 57.3% (282/492) of the participants, fatigue in 54.7% (269/492) of the participants, and influenza-like symptoms in 51% (251/492) of the participants. Younger participants (18-29 years old) exhibited influenza-like symptoms and fever after vaccination in comparison to older participants (P=0.03, P=0.02, respectively). In contrast, older participants reported more immobilization of the hands or legs, difficulty breathing, and chest pain than younger participants (P=0.04, P=0.02, and P=0.03, respectively). There was a significant correlation between gender and injection-site pain, headache, lethargy or convulsions, and flu-like symptoms. In addition, the participants' clinical history of chronic diseases was significantly associated with the occurrence of post-vaccination adverse effects.

Conclusion

The majority of the documented adverse reactions are of a temporary and minimal nature. Additional research is required to assess the enduring adverse reactions and efficacy of the vaccines in preventing SARS-CoV-2 reoccurrence.

## Introduction

The epidemic that was triggered by the SARS-CoV-2 coronavirus is still spreading around the world [1]. The development of a safe and efficient vaccine against SARS-CoV-2 has risen to the top of the list of priorities for medical researchers and practitioners [2]. The development of vaccines generally takes between ten and fifteen years [3]. Vaccines against COVID-19 are being developed at a pace that has never been seen before [4]. Because of the rapid speed of development, there is widespread concern among both the scientific and medical communities, as well as among the general public, that vital phases, such as the assessment of the safety of the vaccine, may have been skipped [4]. The primary objective of vaccination is to protect against diseases in a safe and efficient manner [5]. Pfizer-BioNTech, AstraZeneca Vaxzevria, and Moderna are the three types of vaccines that are currently being used in Saudi Arabia. These vaccines are used because they are readily available. In clinical studies, the effectiveness of the Pfizer-BioNTech vaccine against symptomatic SARS-CoV-2 infection was 95%, while the efficacy of the Oxford-AstraZeneca vaccine was 63% and the efficacy of the Moderna vaccine was 94.1% [6-8]. Nevertheless, in the clinical context, there were some patients who became infected with COVID-19 despite having received the vaccination, while there were other patients who were safeguarded by their prior infection [6-8].

Clinical studies found that each vaccine was linked to a range of side effects that ranged from mild to moderate [6-8]. These effects included feelings of exhaustion, migraines, chills, muscle and joint pains, and a high body temperature [6-8]. This finding was almost exactly the same as those found in investigations carried out in Jordan and Saudi Arabia [9,10]. On the other hand, there is a lack of information regarding the negative impacts over a longer period of time. Concerning the unintended side effects of COVID-19 vaccines, this is an ongoing controversial problem as well as a dynamic circumstance [9]. In spite of the fact that early immunization initiatives were launched in Saudi Arabia, very little is known about the adverse effects of vaccines. The collection of data on the deleterious effects of vaccines that are currently in circulation is absolutely necessary in order to enlighten and educate the general public regarding this matter. The purpose of this study was to establish the incidence of adverse reactions to COVID-19 immunizations provided to the Saudi population in order to develop appropriate preventative measures.

## Materials and methods

This cross-sectional investigation was conducted in Saudi Arabia between October 2021 and October 2022. Most Saudi males and females over the age of 18 who received single, double, or three doses of Pfizer-BioNTech, Oxford-AstraZeneca, or Moderna vaccines were included in the study. We excluded all participants who refused to take part, were unvaccinated against COVID-19, or had received a vaccine manufactured by a company other than Pfizer-BioNTech, Oxford-AstraZeneca, or Moderna.

Data collection instrument

The following three sections were included in the validated dual-language (Arabic and English) online questionnaire used to collect data: sociodemographic information about participants, including age, gender, and disease history (cardiovascular diseases (CVD), diabetes, and endocrine disorders), SARS-CoV-2 infection history, and categories of medications. Information regarding post-vaccination side effects, including the most common short-term side effects (no longer than a week) such as discomfort at injection sites, fatigue, headaches, fever, chills, and muscle and joint pains, as well as long-term side effects (longer than one week). Questions concerning whether any further symptoms, such as SARS-CoV-2 infection, heart attack, or pulmonary embolism, occurred after receiving the COVID-19 vaccination. The participants were selected using a random sampling technique from all regions of Saudi Arabia. For data collection, an online survey was distributed via social media using the Google Platform. Seven hundred forty-five respondents completed the online questionnaire, but 253 responses were omitted from the data set. A final sample of 492 participants was selected for the study.

Ethical statement

The Medical Ethical Committee of the Faculty of Applied Medical Sciences at Taibah University approved this investigation (2021/105/103/MLT). All participants provided informed assent electronically.

Statistical analysis

For the data analysis, we used GraphPad Prism 7 (GraphPad Software, CA, USA). Frequency (percentage) was used to describe categorical statistics and variables. For categorical data analysis, the chi-square test was utilized to evaluate categorical factors such as age, gender, and co-morbidities in relation to adverse reactions following vaccination; P<0.05 indicated statistical significance.

## Results

Baseline characteristics of the study contributors

All 492 participants in this investigation had been immunized against COVID-19. Participants ages ranged from 19 to 29 (53%), 30 to 44 (25%), 45 to 65 (20.7%), and over 65 (1.3%), with a mean age of 35±10.7 years. There were 152 males (30.9%) and 340 females (69.1%). About 32 (25.8%) participants had a history of diabetes, 31 (25%) participants had hypertension, and 20 (16.1%) participants had hypothyroidism. Approximately 8.3% (41/492) of the participants had diseases such as cardiovascular, respiratory, autoimmune, etc. Table 1 shows the main characteristics of the study participants. About 72.2% of study participants received three doses of vaccine: 75.6% received the Pfizer-BioNTech vaccine, 22.2% received the Oxford-AstraZeneca vaccine, and 2.2% received the Moderna vaccine (Table 1). Following vaccination, participants reported fever in 66.3% (326/492) of cases, and headache in 57.3% (282/492). Post-vaccination, 54.7% (269/492) of the participants reported fatigue, while 51% (251/492) reported influenza-like symptoms (Table 2).

**Table 1:** Baseline characteristics of the study contributors (N=492). Numbers (%) are shown. #Geometric mean; SD:∙standard deviation; CVD: cardiovascular diseases; most of the patients suffering from diabetes mellitus, hypertension, and hypothyroidism. §Others included having more than one health problem and those with other diseases such as osteoporosis, gastrointestinal disorders, etc.

Variable	N(%)	
Age (mean±SD)		35±10.7^#^
19-29	261(53%)	
30-44	123(25%)	
45-65	102(20.7%)	
>65	6(1.3%)	
Gender		
Male	152(30.9%)	
Female	340(69.1%)	
Presence of chronic disease		
No chronic disease	368(74.8%)	
Chronic disease		124(25.2%)
Diabetes mellitus	32(25.8%)	
Hypertension	31(25%)	
Hypothyroidism	20(16.1%)	
CVD	8(6.5%)	
Respiratory diseases	17(13.7%)	
Autoimmune	3(2.4%)	
Others^§^	13(10.5%)	
Using medications		
Yes	92(18.7%)	
No	400(81.3%)	
History of COVID infection		
Yes	122(24.8%)	
No	370(75.2%)	
Number of vaccinations		
One dose	8(1.6%)	
Two doses	129(26.2%)	
Three doses	355(72.2%)	
Type of vaccine		
Pfizer-BioNTech	372(75.6%)	
Oxford-AstraZeneca	109(22.2%)	
Moderna	11(2.2%)	

**Table 2:** Post-vaccination short or long-term effects. Numbers (%) are shown for post-vaccination short or long-term effects.

Post-vaccination short-term effects		
	Yes	No
Soreness/redness	150(30.5%)	342(69.5%)
Fever	326(66.3%)	166(33.7%)
Chills	170(34.5%)	322(65.5%)
Headache	282(57.3%)	210(42.7%)
Fatigue	269(54.7%)	223(45.3%)
Flu-like symptoms	251(51%)	241(49%)
Post-vaccination long-term effects		
Headache	55(11.2%)	437(88.8%)
Blurred vision	20(4.1%)	472(95.9%)
Speaking difficulty	5(1%)	487(99%)
Leg swelling	15(3%)	477(97%)
Hand or leg numbness	156(31.7%)	336(68.3%)
Dizziness	44(8.9%)	448(91.1%)
Shortness of breath	87(17.7%)	405(82.3%)
Chest pain	13(2.6%)	479(97.4%)
SARS-CoV-2 infection post-vaccination		
Yes	136(27.6%)	
No	356(72.4%)	
Heart attack		
Yes	6(1.2%)	
No	486(98.8%)	
Pulmonary embolism		
Yes	2(0.4%)	
No	490(99.6%)	

Age and gender comparison of post-vaccination adverse effects

In Tables 3, 4, evaluations of post-vaccination adverse effects and comparisons with demographic characteristics such as age and gender at the time of vaccination are presented. Age was significantly correlated with post-vaccination adverse reactions, such as pain, fever, headache, fatigue, hand or leg numbness, flu-like symptoms, shortness of breath, and chest pain (Table 3). Moreover, younger participants (19-29 years old) were more likely to experience flu-like symptoms and fever after vaccination than elderly participants (P=0.03, P=0.02, respectively). In contrast, older participants reported more hand or leg numbness, shortness of breath, and chest pain than younger participants (P=0.04, P=0.02, P=0.03, respectively). In addition, older participants (45-65 years old) were more likely to experience pain at the injection site, headache, fatigue, and lethargy following vaccination than younger participants (P=0.04, P=0.01, P=0.02, P=0.05, respectively).

**Table 3:** Comparison of post-vaccination side effects with age. Numbers (%) are shown; P-value obtained from chi-square test; *P ≤ 0.05. §Significant differences between 19-29 years old and 45-65 years old.

Post-vaccination side effects	Age (years)								
	19-29=261(53%)		30-44=123(25%)		45-65=102(20.7%)		>65=6(1.2%)		P-value
	Yes	No	Yes	No	Yes	No	Yes	No	
Soreness/redness	60(22.9%)	201(77.1%)	22(17.9%)	101(82.1%)	100(98%)	2(2%)	1(16.7%)	5(83.3%)	0.04*§
Fever	188(72.1%)	73(27.9%)	25(20.4%)	98(79.6%)	50(49.1%)	52(50.9%)	3(50%)	3(50%)	0.02*
Chills	10(3.8%)	251(96.2%)	11(8.9%)	112(91.1%)	15(14.7%)	87(85.3%)	2(33.3%)	4(66.7%)	>0.05
Headache	16(6.1%)	245(93.9%)	5(4.1%)	118(95.9%)	10(9.8%)	92(90.2%)	1(16.7%)	5(83.3%)	>0.05
Fatigue	61(23.4%)	200(76.6%)	23(18.7%)	100(81.3%)	101(99%)	1(1%)	4(66.7%)	2(33.3%)	0.02*§
Flu-like symptoms	189(72.4%)	72(27.6%)	23(18.7%)	100(81.3%)	48(47.1%)	54(52.9%)	3(50%)	3(50%)	0.03*
Headache for a long period	55(21.1%)	206(78.9%)	25(20.3%)	98(79.7%)	99(97.1%)	3(2.9%)	3(50%)	3(50%)	0.01*§
Blurred vision	10(3.8%)	251(96.2%)	11(8.9%)	112(91.1%)	15(14.7%)	87(85.3%)	2(33.3%)	4(66.7%)	>0.05
Speaking difficulty	16(6.1%)	245(93.9%)	5(4.1%)	118(95.9%)	10(9.8%)	92(90.2%)	1(16.7%)	5(83.3%)	>0.05
Leg swelling	10(3.8%)	251(96.2%)	11(8.9%)	112(91.1%)	15(14.7%)	87(85.3%)	2(33.3%)	4(66.7%)	>0.05
Hand or leg numbness	20(7.7%)	241(92.3%)	112(91.1%)	11(8.9%)	99(97.1%)	3(2.9%)	5(83.3%)	1(16.7%)	0.04*
Dizziness	45(17.2%)	216(82.8%)	30(24.4%)	93(75.6%)	96(94.1%)	6(5.9%)	1(16.7%)	5(83.3%)	0.05*§
Shortness of breath	18(6.9%)	243(93.1%)	115(93.5%)	8(6.5%)	98(96.1%)	4(3.9%)	4(66.6%)	2(33.4%)	0.02*
Chest pain	13(4.9%)	248(95.1%)	117(95.1%)	6(4.9%)	97(95.1%)	5(4.9%)	6(100%)	0(0%)	0.05*

Gender was shown to be substantially associated with injection-site pain, headaches, dizziness, and influenza-like symptoms. Females were more likely to experience injection-site pain (P=0.02), fever (P=0.01), headache (P=0.03), influenza-like symptoms (P=0.02), and dizziness (P=0.02). Post-vaccination, males were more likely to experience hand or leg numbness (P=0.04) and shortness of breath (P=0.05) (Table 4).

**Table 4:** Comparison of post-vaccination side effects with gender. Numbers and percentages are shown; P-value obtained from chi-square test; *P ≤ 0.05.

Post-vaccination side effects	Male=152(30.9%)		Female=340(69.1%)		P-value
	Yes	No	Yes	No	
Soreness/redness	52(34.2%)	100(65.8%)	200(58.8%)	140(41.2%)	0.02*
Fever	25(16.4%)	127(83.6%)	160(47.1%)	180(52.9%)	0.01*
Chills	50(32.9%)	102(67.1%)	55(16.2%)	285(83.8%)	>0.05
Headache	54(35.5%)	98(64.5%)	166(48.8%)	174(51.2%)	0.03*
Fatigue	100(65.8%)	52(34.2%)	255(75%)	85(25%)	>0.05
Flu-like symptoms	60(39.5%)	92(60.5%)	214(62.9%)	126(37.1%)	0.02*
Blurred vision	3(2%)	149(98%)	5(1.5%)	335(98.5%)	>0.05
Speaking difficulty	2(1.3%)	150(98.7%)	2(0.6%)	338(99.4%)	>0.05
Leg swelling	3(2%)	149(98%)	2(0.6%)	338(99.4%)	>0.05
Hand or leg numbness	70(46.1%)	82(53.9%)	16(4.7%)	324(95.3%)	0.04*
Dizziness	20(13.2%)	132(86.8%)	33(9.7%)	307(90.3%)	0.02*
Shortness of breath	74(48.7%)	78(51.3%)	54(15.9%)	286(84.1%)	0.05*
Chest pain	14(9.2%)	138(90.8%)	19(5.6%)	321(94.4%)	>0.05

Comparative analysis of post-vaccination adverse events and comorbidities

The clinical history of chronic diseases was also significantly related to the development of post-vaccination adverse reactions (Table 5). After vaccination, diabetic participants were more likely to experience fatigue (P=0.04), influenza-like symptoms (P=0.03), and headaches (P=0.05). Similarly, hypertensive participants were more likely to experience hand or leg numbness after injection (P=0.04) and shortness of breath after vaccination (P=0.03). Also, hypothyroidism patients more commonly had fatigue, flu-like symptoms, and headaches, in addition to chest pain post-vaccination (all P<0.05, Table 5).

**Table 5:** Comparison of post-vaccination side effects with comorbidities. Numbers and percentages are shown.

Post-vaccination side effects	Diabetes=32(25.8%)		Hypertension=31(25%)		Hypothyroidism=20(16.1%)	
	Yes	No	Yes	No	Yes	No
Soreness/redness	16(50%)	16(50%)	17(54.8%)	14(45.2%)	10(50%)	10(50%)
Fever	18(56.2%)	14(43.8%)	15(48.4%)	16(51.6%)	9(45%)	11(55%)
Chills	17(53.1%)	15(46.9%)	18(58.1%)	13(41.9%)	10(50%)	10(50%)
Fatigue	30(93.8%)	2(6.2%)	30(96.7%)	1(3.3%)	15(75%)	5(25%)
Flu-like symptoms	31(96.9%)	1(3.1%)	29(93.5%)	2(6.5%)	17(85%)	3(15%)
Blurred vision	16(50%)	16(50%)	17(54.8%)	14(45.2%)	10(50%)	10(50%)
Speaking difficulty	18(56.2%)	14(43.8%)	15(48.4%)	16(51.6%)	9(45%)	11(55%)
Leg swelling	17(53.1%)	15(46.9%)	18(58.1%)	13(41.9%)	10(50%)	10(50%)
Hand or leg numbness	30(93.8%)	2(6.2%)	28(90.3%)	3(9.7%)	11(55%)	9(45%)
Dizziness	17(53.1%)	15(46.9%)	15(48.4%)	16(51.6%)	9(45%)	11(55%)
Shortness of breath	29(90.6%)	3(9.4%)	15(48.4%)	16(51.6%)	18(90%)	2(10%)
Chest pain	17(53.1%)	15(46.9%)	15(48.4%)	16(51.6%)	18(90%)	2(10%)

## Discussion

Initial research conducted in a variety of countries, including France, Russia, and Poland, reveals that the inhabitants of those countries exhibit a high degree of resistance to receiving vaccinations [11,12]. On the other hand, a large number of studies have shed light on the significance of COVID-19 immunization in lowering rates of fatalities and hospitalizations [13].

It is imperative that a COVID-19 vaccination that is both safe and efficient be given to our population in order to protect them from the potentially devastating effects that the COVID-19 pandemic could have on humankind. According to the findings of the current research, transient and limited deleterious effects are prevalent with immunizations. These effects can also differ depending on the gender, age, and medical history of the participants. In this particular research, the Pfizer-BioNTech vaccine was the one that was found to be the most widespread and was positively accepted by participants of all ages (19-65 years) with no significant adverse effects. The post-vaccination adverse effects that were experienced by most people were headaches, fatigue, fever, and symptoms similar to the flu. Some side effects, such as numbness in the hands or limbs, shortness of breath, and chest pain, were reported in older age groups but not in younger age groups, suggesting a direct association between age and these categories of side effects. No side effects were reported in the younger age groups. In addition, there was a correlation between gender and the categories of adverse effects; for example, female participants experienced soreness at the injection site as well as fatigue or dizziness, whereas male participants experienced hand or limb numbness and shortness of breath. Male participants also experienced a correlation between gender and the categories of adverse effects. Research that was carried out in Iraq in 2022 found similar findings, suggesting that females were more susceptible to the deleterious effects of COVID-19 than males due to hormonal and physiological variables [14].

In addition, it was discovered that a significant number of participants in the current study who had a medical history of comorbidities such as diabetes, hypertension, or hypothyroidism were more likely to have fatigue, flu-like symptoms, headaches, and chest pain after receiving the vaccination. Our conclusions are in line with those of other studies carried out in different parts of the world, including but not limited to Europe, the United States of America, the United Kingdom, Australia, and China [15-17]. A clinical experiment and an evaluation of the adverse effects were carried out by AstraZeneca in the United Kingdom. A significant number of the people who took part in the study reported experiencing fever, exhaustion, headaches, and muscular pain [12]. Following vaccination, additional research that was carried out in the United States by BioNTech-Pfizer found that 83.3%, 100%, 58.7%, and 66.7% of the participants encountered fatigue, headaches, and muscular discomfort, respectively [13,14]. On the other hand, there is a possibility that the side effects will change depending on the population and the surroundings, and the type of vaccine is also a significant component in determining the severity of the side effects [18]. However, one patient was hospitalized following the COVID-19 vaccination by Johnson & Johnson, which increased requests for diagnosing cases shortly after vaccination because some trials had transient neutropenia and lymphopenia after vaccination. The current study found that the side effects of the COVID-19 vaccine were generally mild, which is consistent with the findings of the previous study [15-19].

In Saudi Arabia, there is a lack of information regarding the possible negative side effects of COVID-19 immunization. The COVID-19 vaccine is met with a great deal of skepticism from people all over the world due to the widespread belief that it could make the illness more contagious and reduce its efficiency as a prophylactic measure. Because of this, the fact that the COVID-19 vaccine has very few adverse effects, as explained in our research, will help people feel less anxious or fearful about receiving the vaccine. In addition, a vaccine that lowers the total number of cases would probably be approved at the population level first and foremost to reduce the chance of unintended side effects. In addition, scientists from all over the world have used a wide range of tools to make and improve a vaccine that can reduce the harmful effects of infection. This has been shown in a number of studies [16-20] that looked at how well vaccines worked and how safe they were. Even so, accepted and approved vaccines will continue to face problems, and because the vaccines are new, the general public may be skeptical about how well they work. Literature reports say that 82% of a region's population should be vaccinated to increase "herd immunity." However, introductory literature reports show that there is a lot of skepticism about vaccines, which experts acknowledge. Furthermore, participants in the current study haven't reported any serious side effects, and 72.2% of them have gotten three doses of the vaccine without any major side effects. This suggests that this may help lessen people's resistance to getting the vaccine.

However, current research has some limitations. First, it is cross-sectional, limited to a single geographical location (primarily the Madinah Province), and it is challenging to determine cause-and-effect relationships. The pilot study was conducted shortly after the Saudi Arabian vaccination campaign began for the general population. Therefore, as more data, evidence, and information become available on the safety and efficacy of COVID-19 vaccines, individuals' attitudes toward vaccination may shift. Despite the above limitations, our pilot study is one of the few to compare COVID-19 vaccine adverse effects in Saudi Arabia.

## Conclusions

The data presented indicates that the majority of adverse effects associated with vaccines are of a temporary and minor nature. The most commonly observed adverse events included headache, fatigue, fever, and flu-like symptoms, which contain resemblance to those reported in vaccine trials. The variability of side effects is dependent upon factors such as age, gender, and medical history of the participants. Additional research is required to examine the efficacy of existing vaccines in preventing SARS-CoV-2 reinfection. In addition, it is necessary to assess the potential causal relationship between vaccines and the reported adverse effects that persist over an extended period of time.
